# Bioactive Properties of *Syzygium cumini* (L.) Skeels Pulp and Seed Phenolic Extracts

**DOI:** 10.3389/fmicb.2020.00990

**Published:** 2020-05-27

**Authors:** Catarina A. Santos, Felipe A. Almeida, Beatriz X. V. Quecán, Patricia A. P. Pereira, Kelly M. B. Gandra, Luciana R. Cunha, Uelinton M. Pinto

**Affiliations:** ^1^Faculty of Pharmaceutical Sciences, Food Research Center, University of São Paulo, São Paulo, Brazil; ^2^Department of Nutrition, Federal University of Juiz de Fora, Governador Valadares, Brazil; ^3^Department of Foods, Federal University of Ouro Preto, Ouro Preto, Brazil

**Keywords:** antimicrobial compounds, biofilm, antioxidant, quorum sensing, phenolic compounds

## Abstract

The emergence of bacterial strains resistant to different antibiotics has prompted the search for new sources of antimicrobial compounds. Studies have shown that jambolan [*Syzygium cumini* (L.) Skeels], a tropical fruit from the Mirtaceae family, contains a great variety of phytochemical compounds with high antioxidant and antimicrobial activity. This study aimed to determine the centesimal composition and physicochemical characteristics of the pulp and seed of *S. cumini* (L.) Skeels, as well as the content of total phenolic compounds and the antioxidant, antibacterial, antibiofilm and anti-quorum sensing (QS) activities of the phenolic extracts obtained from the pulp and the seeds of this fruit. The *in vitro* antibacterial and anti-QS activities of active films incorporating phenolic extracts were also evaluated. Additionally, we performed molecular docking of phenolic compounds present in jambolan with the CviR QS regulator of *Chromobacterium violaceum*. The composition and physicochemical characteristics of the samples presented similar values to those found for the species. However, the seed phenolic extract had a higher content of phenolic compounds and antioxidant activity than the pulp. Both phenolic extracts presented antibacterial activity against *Aeromonas hydrophila*, *C. violaceum*, *Escherichia coli*, *Pseudomonas aeruginosa*, *Salmonella enterica* serovar Typhimurium, *Serratia marcescens*, *Listeria monocytogenes*, and *Staphylococcus aureus*. The seed phenolic extract was particularly inhibitory against *S. aureus.* The pulp phenolic extract inhibited swarming motility and biofilm formation of *A. hydrophila*, *E. coli*, and *S. marcescens* in sub-MIC concentrations. The pulp and seed phenolic extracts inhibited violacein production in *C. violaceum*. Films incorporating both phenolic extracts inhibited the growth of bacteria, particularly *Pseudomonas fluorescens, L. monocytogenes*, and *S. aureus*, as well as QS in *C. violaceum*. Molecular docking showed that a variety of compounds found in pulp and seed extracts of jambolan, particularly chlorogenic acid and dihydroquercetin, potentially bind CviR protein and may interfere with QS. Our results indicate that pulp and seed of jambolan are good sources of antibacterial, antibiofilm, and anti-QS compounds that can be used in the development of natural preservatives and for application in antibacterial active films.

## Introduction

*Syzygium cumini* (L.) Skeels, known as jambolan or by its several popular names in Brazil, black plum, northeastern olive, nun berry, and earth olive, belongs to the Myrtaceae family. It is native to India, Ceylon, Malaysia, and Australia and is also found in several regions in Brazil, fruiting from January to May (Vizzotto and Pereira, 2008). Although jambolan is commonly found in Brazil, consumption of its fruits is scarce, and the plant is mostly unknown by the general population. In India and Pakistan, this fruit is widely used in folk medicine (Loguercio et al., 2005).

*Syzygium cumini* (L.) Skeels presents a high antioxidant activity and high levels of phenolic compounds, which have a protective effect on food, mainly because of its antioxidant properties (Tavares et al., 2017). Antioxidants can delay the oxidation of different substrates, working as radical hijackers and sometimes as metal chelators (Dannenberg et al., 2016). In addition, the antioxidants may also present antimicrobial activity (Loguercio et al., 2005). Moreover, several studies using leaves, bark, essential oils, fruits, and seeds have demonstrated the beneficial effects of jambolan, such as antioxidant (Vizzotto and Pereira, 2008; Singh et al., 2016) and antimicrobial activities (Singh et al., 2016). However, nothing is known about the inhibition of bacterial quorum sensing (QS) by *S. cumini* (L.) Skeels.

Many bacteria use cell-to-cell communication as a means of assimilating environmental cues, communicating with each other, and monitoring their own population density in order to modulate gene expression. This bacterial density-dependent communication system is termed QS. The increase in bacterial population leads to the accumulation of signaling molecules that are necessary for activation of specific receptors. The phenotypes regulated by QS involve motility, biofilm formation, production of violacein, and resistance to antibiotics, among others (LaSarre and Federle, 2013; Rivera et al., 2019). QS regulates several phenotypes that contribute to virulence and can also affect food safety and spoilage phenotypes.

The emergence of antibiotic-resistant bacterial strains encourages the search for new sources of antimicrobial and anti-QS compounds. The antimicrobial properties of substances extracted from plants have been extensively studied, and most of the active compounds of medicinal plants are conferred by products of the secondary metabolism (Lin et al., 2016; Rivera et al., 2019). Among these substances, phenolic compounds stand out for having different properties, such as antioxidant, antimicrobial, anti-inflammatory, and antiproliferative activities (Manach et al., 2004; Tortora et al., 2012; Xu et al., 2014).

The use of active films incorporating antimicrobial agents as primary packaging, which are in direct contact with the food, is an option for the direct application of raw extracts to foods, because in addition to reducing unwanted interference and protecting sensitive bioactive compounds, it allows the release of the active components at the surface of the food, where contamination usually takes place (Vonasek et al., 2014). In addition, the lack of reports in this area reinforces the importance of studying films incorporating extracts of pulp and seeds of jambolan, exploring their potential to improve safety in food packaging.

This study aimed to determine the centesimal composition and physicochemical characteristics of pulp and seed of jambolan, as well as the content of total phenolic compounds and the antioxidant, antibacterial, antibiofilm, and anti-QS activities of the phenolic extracts obtained from its seeds and pulp. In addition, we evaluated the *in vitro* antibacterial and anti-QS activities of active films incorporating these phenolic extracts, besides performing *in silico* analysis with phenolic compounds found in jamboaln and the QS CviR protein of *C. violaceum*.

## Materials and Methods

### Pulp and Seed of *S. cumini* (L.) Skeels

Initially, the characteristics of the tree of *S. cumini* (L.) Skeels located in the rural area of the town of Viçosa, Minas Gerais, Brazil (coordinates 20° 45’ 17” S, 42° 52’ 57” W) were compared with those described for *Syzygium cumini* (L.) Skeels by Tavares (2015) of Paulista State University (UNESP), São José do Rio Preto, Brazil (coordinates 20° 49′ 12″ S, 49° 22′ 12″ W, barcode SJRP00008916) deposited at Reflora – Herbário Virtual^1^. Jambolan fruits were collected at the fruiting season, between January and May 2016, in the mature stage. The fruits were washed and immersed in a sodium hypochlorite solution 0.005% (w/v) for 15 min (Oliveira et al., 2016). Subsequently, pulp and seeds were manually separated and stored at −80°C until analysis. Before freezing, centesimal composition and physicochemical characteristics of pulp and seed of *S. cumini* (L.) Skeels were determined.

#### Moisture

Moisture was determined from fresh pulp and seed according to the method described by the Association of Official Analytical Chemists (AOAC, 2005). Pulp samples were mashed and homogenized in a vessel, and the seed was crushed in an industrial blender. An aliquot of 8 g of both samples was weighed in a porcelain capsule. Samples were heated at 105°C for 3 h, and, after being cooled in a desiccator, they were weighed. Heating and cooling operation was repeated until constant weight. The percentage of moisture was calculated following the formula, where N is the weight in grams of moisture and P is the total weight of the sample.

(1) $$\left(\%\right)moisture=\frac{N}{P}\times 100$$

#### Ashes

Ash determination was carried out from fresh pulp and seed according to the Association of Official Analytical Chemists (AOAC, 2005). From the kneaded and homogenized samples, 8 g were weighed in a porcelain container previously heated in a muffle furnace at 550°C, cooled in a desiccator at room temperature and weighed. The samples were incinerated in a muffle at 550°C, until complete elimination of organic matter. When the ash became white or slightly gray, the samples were cooled in a desiccator to room temperature and weighed. The percentage of ash was calculated according to formula 1, where N is the weight in grams of ash and P is the total weight of the sample.

#### Proteins

Protein determination was performed by the Kjeldahl digestion process according to the Association of Official Analytical Chemists (AOAC, 2005) with 1 g of dry sample from the moisture test (section “Moisture”) and transferred to the Kjeldahl tube. The percentage of proteins was calculated according to formula 2, where V is the volume of 0.05 M sulfuric acid spent on the titration, P the total sample weight, and f is the conversion factor (=6.25).

(2) $$\%\,p\,r\,o\,t\,e\,i\,n\,s=\frac{V\times 0.14\times f}{\text{P}}\times 100$$

#### Lipids

Quantification of lipids was performed by the Soxhlet process according to the Association of Official Analytical Chemists (AOAC, 2005). An aliquot of 5 g of the dried samples was weighed from the moisture test (section “Moisture”) and transferred to the Soxhlet cartridge. This was coupled to the Soxhlet type extractor, and to a flat-bottomed flask previously set at 105°C. Then, ether was added in sufficient amount to fill the Soxhlet extractor, and extraction was performed for 8 h under electric plate heating. Later, the cartridge was removed and the flask with the extracted residue was placed in a stove at 105°C for an hour. After cooling in a desiccator, the bottles were weighed. The percentage of lipids was calculated following formula 1, where N is the weight in grams of lipids and P is the total weight of the sample.

#### Carbohydrates

The total carbohydrates were calculated by the difference of the value obtained by the sum of moisture, ashes, proteins, and lipids.

#### Total Soluble Solids

Total soluble solids were evaluated using a portable digital refractometer (Instrutherm, Brazil).

#### Titratable Acidity and pH

Titratable acidity was determined by the volumetric technique with an indicator, according to Instituto Adolfo Lutz (2008). The samples were pressed in a crucible and diluted in an Erlenmeyer flask with 100 mL of distilled water. Phenolphthalein indicator was added and titrated with 0.1 M sodium hydroxide solution under constant agitation, until a persistent pink color was observed. The percentage of titratable acidity was calculated following formula 3, where V is the volume of the hydroxide solution spent on the titration, f is the correction factor for the sodium hydroxide solution, P is the total mass of the sample, and M the molarity of the sodium hydroxide solution.

(3) $$\left(\%\right)titratableacidity=\frac{V\times f\times M}{\text{P}}\times 100$$

The pH was evaluated by measuring in a digital pH meter (Digimed, Brazil).

### Pulp and Seed Phenolic Extracts of *S. cumini* (L.) Skeels

#### Extraction of Phenolic Compounds of Pulp and Seed of *S. cumini* (L.) Skeels

Samples of pulp and seed jambolan were thawed, ground in an industrial blender and homogenized with ethanol:methanol:acetone solution (1:1:1:1, g/v/v/v) for extraction of phenolic compounds. The mixture was vacuum filtered on Whatman paper 1, and the organic solvents were evaporated at 40°C in a rotary evaporator (Buchi, Brazil), obtaining the pulp and seed phenolic extracts of *S. cumini* (L.) Skeels in water. The phenolic extracts obtained were stored in sterile amber glass for protection against light and stored at −80°C. The expression “phenolic extract” used in the text has no relation to the solvents used to obtain the extract.

#### Content of Total Phenolic Compounds

The total phenolic compounds of the pulp and seed phenolic extracts was determined by the Folin-Ciocalteau assay, with modifications (Waterhouse, 2002). An aliquot of 500 μL of phenolic extract or ethanol (blank control) was placed in a test tube with 2.5 mL of Folin-Ciocalteau reagent (10% v/v). Subsequently, 2.0 mL of saturated sodium carbonate solution (4% w/v) was added. After 2 h, the absorbance was determined at 750 nm by spectrophotometer (UV-1601 PC Shimadzu, Japan). The total phenolic content was determined using the standard curve of gallic acid. The results were expressed as mg of gallic acid equivalent per grams of pulp or seed (mg GAE/g of pulp or seed).

#### Antioxidant Activity of Phenolic Extracts

The antioxidant activity of the pulp and seed phenolic extracts was determined by the DPPH (2,2-diphenyl-1-picrylhydrazyl) method (Rufino et al., 2010). The standard curve was prepared with DPPH solutions at different concentrations such as 10, 20, 30, 40, 50, and 60 μM. From the obtained absorbances, the equation of the line was determined in order to determine the EC_50_ in g of fresh sample/g of DPPH. The EC_50_ corresponds to the sample concentration required to reduce the initial DPPH radical concentration by 50%. All analyses were performed protected from light.

#### Bacterial Strains and Their Growth Conditions

*Aeromonas hydrophila* IOC/FDA 110-36, *Escherichia coli* ATCC 10536, *Pseudomonas aeruginosa* ATCC 15442, *Salmonella enterica* serovar Typhimurium ATCC 14028, *Listeria monocytogenes* ATCC 7644 and *Staphylococcus aureus* ATCC 6538P were grown at 37°C for 18 h, and *Chromobacterium violaceum* ATCC 12472, *Serratia marcescens* RM1 (a strain isolated from raw milk – laboratory stock) and *Pseudomonas fluorescens* NCTC 10038 were grown at 30°C for 18 h. Antibacterial activity tests were performed in Brain Heart Infusion (BHI) medium, and anti-QS activity tests were performed in Luria-Bertani (LB) medium for pulp and seed phenolic extracts and active films incorporating these extracts.

#### Antibacterial Activity of Phenolic Extracts

The antibacterial activity of the pulp and seed phenolic extracts was performed by agar diffusion assay and by liquid diffusion assay, which included minimum inhibitory concentration (MIC) and inhibitory potential (IP) tests. The phenolic extracts were tested at concentrations of up to 0.78 mg GAE/g of pulp and at concentrations of up to 11.29 mg GAE/g of seed.

##### Agar diffusion assay

The agar diffusion assay was performed according to the method described by Salawu et al. (2011) with modifications. A volume of 20 mL of BHI agar was inoculated with 10^8^ CFU/mL of each microorganism and poured into sterile Petri dishes. After solidification, 5 mm holes were made with the aid of sterile tips, and aliquots of 20 μL of extract were added to each hole. The plates were kept refrigerated for 18 h and subsequently incubated for 24 h. The inhibitory activity of the extract was verified through the formation of clear zones around the holes, compared to the control (sterile water) and expressed by the average (triplicate) of the diameter (mm) of the inhibition halos.

##### Minimum inhibitory concentration (MIC)

Determination of the minimum inhibitory concentration (MIC) was performed according to described by Wiegand et al. (2008) with modifications. BHI broth (300 μL) containing different concentrations of crude pulp and seed extract was added to 1.5-mL sterile microtubes. Subsequently, each microtube was inoculated with 10^5^ CFU/mL of the test microorganism, grown overnight. The microtube was incubated for 24 h. The minimum inhibitory concentration (MIC) was considered as the lowest concentration of extract in which there was no visible bacterial growth.

##### Inhibitory potential (IP)

The inhibitory potential (IP) was performed according to the method described by Alvarez et al. (2012) with modifications. Volumes of 300 μL of BHI broth containing different concentrations of crude pulp and seed extract were added to the microtube. Subsequently, each microtube was inoculated with 3 μL of the test microorganism containing 10^8^ CFU/mL in BHI broth. Incubation was carried out for 24 h. Subsequently, serial dilution of the samples and plating (surface) on BHI agar was performed. The plates were incubated in the same conditions as described above, and the CFUs were counted. The relative results were expressed using the IP, calculated according to the formula described in equation 4, where No represents the count in CFU/mL of the control sample (0% of extract) and N the count in CFU/mL of the sample in the test concentration. An IP equal to 1 indicates 10-times inhibition, and a twofold inhibition gives an IP of 0.301.

(4) $$I\,P=\text{log}\,\left(\frac{N\,o}{N}\right)$$

#### Anti-QS Activity of Phenolic Extracts

The anti-QS activity of the pulp and seed phenolic extracts in sub-minimum inhibitory concentration (sub-MIC) was evaluated against QS regulated phenotypes.

##### Swarming motility

The test was performed according to Packiavathy et al. (2012). In a volume of 3 mL of semi-solid LB agar 0.5% (w/v), the pulp phenolic extract was added at sub-MIC of 0.19 and 0.39 mg GAE/g of pulp, which is equivalent to 12.5 and 25% (v/v) of phenolic extract, respectively. Subsequently, the tubes were homogenized in vortex and transferred into small Petri dishes (49 mm × 12 mm). After 10 min, 2 μL of inoculum containing 10^8^ CFU/mL of *A. hydrophila* or *S. marcescens* were inoculated at the center of the plate, followed by 24 h incubation and subsequent measurement of the diameter of the colony. Semi-solid agar without extract was used as control.

##### Biofilm formation

The analysis was performed according to Trentin et al. (2011). Aliquots of 200 μL of LB broth were added with pulp phenolic extract of *S. cumini* (L.) Skeels in sub-MIC of 0.19, 0.39, and 0.78 mg GAE/g of pulp. Aliquots of the 2 μL of inoculum containing 10^5^ CFU/mL of *A. hydrophila* or *E. coli* or *S. marcescens* were inoculated and incubated for 24 h at the optimal growth temperature of each bacterium (30°C or 37°C). After incubation, the optical density was measured at 600 nm and planktonic cells were removed by turning the microplate over into absorbent paper. The sessile cells were stained with 200 μL of 0.1% (w/v) of crystal violet for 30 min, the dye was removed, and the wells rinsed with distilled water three times. The plates were dried at 40°C for 15 min. The crystal violet retained by the adhered cells was dissolved in 200 μL of ethanol (95% v/v), and the absorbance at 630 nm was determined by spectrophotometer (UV1601 PC Shimadzu, Japan). The results of the biofilm formation were obtained according to equation 5:

(5) $$\%\mathrm{biofilm}\mathrm{formation}=\frac{\left(\text{ODtest}-\mathrm{ODbroth}\,\mathrm{extract}\right)}{\left(\text{ODcontrol}-\text{ODbroth}\right)}\times 100$$

In which: OD, optical density; Test, LB broth with phenolic extract and bacteria; Broth Extract, blank LB broth with phenolic extrac; Control, LB broth with bacteria; Broth, blank LB broth.

##### Violacein production

Quantification of violacein production was performed according to Quecán et al. (2019) with modifications. The assay was performed in tubes containing 2 mL of LB broth and 200 μL of inoculum containing 10^6^ CFU/mL of *C. violaceum* with pulp and seed phenolic extracts at sub-MIC of 0.10 mg GAE/g of pulp and 0.04 mg GAE/g of seed, respectively. The tubes were incubated for 24 h at 30°C and 150 rpm. Subsequently, 200 μL of the culture from each test was placed on a 96-well plate and the OD_600_ was measured. In addition, 1 mL of the culture from each test was placed in microtubes and centrifuged at 13000 rpm for 10 min in order to precipitate insoluble violacein. The supernatant was discarded, and the pellet was solubilized in 1 mL of dimethyl sulfoxide (DMSO). This pellet was mixed and centrifuged under the same conditions described above, and the supernatant was subsequently collected to perform the quantification of violacein. The absorbance was measured on a 96-well plate at 585 nm by spectrophotometer. LB broth without extract was used as negative control, and the positive control for inhibition of QS was performed with addition of 39.4 μM of 4-bromo-5-(bromomethylene)-2(5H)-furanone (Furanone C30; Sigma-Aldrich, Brazil) as described by Oliveira et al. (2016). The percentage of violacein production was calculated by means of the formula used for biofilm formation.

### Active Films Incorporating Pulp and Seed Phenolic Extracts of *S. cumini* (L.) Skeels

#### Production of Active Films Incorporating Phenolic Extracts

The films were produced by the casting method using cellulose acetate as a polymeric matrix according to Soares et al. (2008). An aliquot of 10 mL of filmogenic solution was formed by cellulose acetate in acetone (1% w/v), and after complete polymerization, 1 mL of each pulp or seed phenolic extract was added separately. Subsequently, the solution was deposited on a sterile flat surface for film formation and evaporation of acetone at room temperature. Then, the films were cut in an area of 3.14 cm^2^ considering both sides, stored in sterile Petri dishes and light-protected. The control films without the phenolic extracts or with 39.4 μM of furanone C30 were produced separately.

#### *In vitro* Antibacterial Activity of Active Films Incorporating Phenolic Extracts

The antibacterial activity of two 2-cm diameter discs of films incorporating phenolic extracts of pulp or seed, furanone C30 or with no additions was performed by IP in BHI broth against *E. coli*, *P. aeruginosa*, *Salmonella* Typhimurium, *L. monocytogenes*, and *S. aureus*, as described in section “Inhibitory potential (IP).”

#### *In vitro* Anti-QS Activity of Active Films

##### Violacein production

Quantification of violacein production was performed according to Quecán et al. (2019) with modifications described in section “Violacein production.” In addition, tubes were used containing 3 mL of LB broth, 300 μL of inoculum containing 10^6^ CFU/mL of *C. violaceum*, and two 2-cm diameter discs of films either incorporating phenolic extracts of pulp, seed, or furanone C30 or with no additions.

### *In silico* Analysis of Compounds Found in Pulp and Seed Extracts of *S. cumini* (L.) Skeels

#### Molecular Docking With Structures of CviR Protein of *C. violaceum* ATCC 12472

Docking studies were performed according to Almeida et al. (2018) to evaluate the potential anti-QS activity of compounds found in pulp and seed extracts of *S. cumini* (L.) Skeels described in the literature. The 3QP6 and 3QP8 crystallized structures of CviR protein of *C. violaceum* ATCC 12472 with *N*-hexanoyl-DL-homoserine lactone (C6-HSL) and *N*-decanoyl-DL-homoserine lactone (C10-HSL), respectively (Chen et al., 2011), were obtained from the RCSB Protein Data Bank database (PDB)^2^. Then, the molecular docking was performed between these proteins and *N*-(3-hydroxydecanoyl)-DL-homoserine lactone (3-OH-C10-HSL; PubChem CID: 71353010), C10-HSL (PubChem CID: 11644562), C6-HSL (PubChem CID: 3462373), 4-bromo-5-(bromomethylene)-2(5H)-furanone (Furanone C30; PubChem CID: 10131246) and 47 phenolic compounds found in pulp and seed extracts of *S. cumini* (L.) Skeels with structure deposited in the PubChem using the “Dock Ligands” tool of the CLC Drug Discovery Workbench 4.0 software^3^, with 1000 interactions for each compound, and the conformation of the compounds was changed during the docking via rotation around flexible bonds. The generated score mimics the potential energy change when the protein and the compound interact based on hydrogen bonds, metal ions, and steric interactions, where lower scores (more negative) correspond to higher binding affinities. The five best scores of the docking of each compound were selected, allowing the inspection of the binding sites of CviR protein with each compound (Almeida et al., 2018).

### Statistical Analyses

Experiments were performed in three biological replicates. The values of the triplicates were used for the analysis of variance (ANOVA) followed by Tukey’s test using the Statistical Analysis System and Genetics Software (Ferreira, 2011). A *p*-value *<* 0.05 was statistically significant.

## Results

### Centesimal Composition and Physicochemical Characteristics of Pulp and Seed of *S. cumini* (L.) Skeels

Table 1 presents the results obtained for the centesimal composition and physicochemical characteristics of the pulp and seed of *S. cumini* (L.) Skeels. The pulp had higher moisture and ash content than the seed (*p* < 0.05) and lower content of proteins, lipids, and carbohydrates, as well as titratable acidity and pH (*p* < 0.05) (Table 1).

**TABLE 1:** Centesimal composition, physicochemical characteristics, content of total phenolic compounds, and antioxidant activity of the pulp and seed of *S. cumini* (L.) Skeels.

**Component (Unit)**	**Pulp (M ± SD)**	**Seed (M ± SD)**
**Fruit**		
Moisture (%)	83.51 ± 0.05^a^	62.25 ± 6.32^b^
Ashes (%)	1.38 ± 0.19^a^	0.36 ± 0.02^b^
Proteins (%)	5.62 ± 0.44^b^	19.96 ± 0.00^a^
Lipids (%)	0.97 ± 0.14^b^	2.47 ± 0.24^a^
Carbohydrates (%)	8.52 ± 0.65^b^	14.95 ± 6.09^a^
Total soluble solids (°Brix)	12.93 ± 0.06	n.d.
Titratable acidity (%)	0.65 ± 0.03^b^	0.87 ± 0.03^a^
pH	4.12 ± 0.01^b^	4.83 ± 0.03^a^
**Phenolic extract**		
Content of total phenolic compounds (mg GAE/g of pulp or seed)	1.56 ± 0.01^b^	22.59 ± 0.79^a^
Antioxidant activity (EC_50_ g of fresh sample/g of DPPH)	5301.95 ± 96.28^a^	211.75 ± 10.99^b^

*M ± SD, Means ± standard deviation, Means followed by different superscript letters in the same line differ among themselves at 5% probability (p ¡ 0.05) by Tukey’s test. n.d., not determined; GAE, gallic acid equivalent; EC_50_, corresponds to the sample concentration required to reduce the initial DPPH radical concentration by 50%. DPPH – (2,2-diphenyl-1-picrylhydrazyl).*

### Pulp and Seed Phenolic Extracts of *S. cumini* (L.) Skeels

#### Content of Total Phenolic Compounds and Antioxidant Activity of Phenolic Extracts

Table 1 also presents the content of total phenolic compounds and antioxidant activity of the pulp and seed phenolic extracts of *S. cumini* (L.) Skeels. The jambolan seed phenolic extract had a higher content of phenolic compounds than the pulp (*p* < 0.05), as well as a remarkable higher antioxidant activity (*p* < 0.05) (Table 1).

#### Antibacterial Activity of Phenolic Extracts

##### Agar diffusion assay

In this assay, only *S. aureus* was inhibited by 22.59 mg GAE/g of seed extract of jambolan, forming an inhibition zone of 24.5 ± 0.07 mm in diameter. However, this bacterium was not inhibited by the pulp phenolic extract in this setting. On the other hand, neither the pulp phenolic extract nor the seed phenolic extract inhibited *E. coli*, *P. aeruginosa*, *Salmonella* Typhimurium and *L. monocytogenes* in this assay.

##### Minimum inhibitory concentration (MIC)

Table 2 shows the MIC of the pulp and seed phenolic extracts of *S. cumini* (L.) Skeels against the evaluated bacteria. The seed phenolic extract was more effective at inhibiting the evaluated bacteria than the pulp phenolic extract (Table 2). The MIC of the pulp phenolic extract was greater than 0.78 mg GAE/g of pulp for all the evaluated bacteria (1:2 extract dilution), except for *C. violaceum* in which the MIC was 0.39 mg GAE/g of pulp (1:4 extract dilution) (Table 2). On the other hand, the MIC values of seed phenolic extracts varied from 0.09 to 11.29 mg GAE/g of seed (dilutions ranging from 1:2 to 1:256) (Table 2).

**TABLE 2:** Minimum inhibitory concentration (MIC) of the pulp and seed phenolic extracts of *S. cumini* (L.) Skeels against the evaluated bacteria.

**Bacteria**	**Phenolic extract**			
	**Pulp**		**Seed**	
	**Concentration (mg GAE/g of pulp)**	**Extract dilution**	**Concentration (mg GAE/g of seed)**	**Extract dilution**
*A. hydrophila*	>0.78	1:2	11.29	1:2
*C. violaceum*	0.39	1:4	0.09	1:256
*E. coli*	>0.78	1:2	11.29	1:2
*P. aeruginosa*	>0.78	1:2	11.29	1:2
*Salmonella* Typhimurium	>0.78	1:2	5.65	1:4
*S. marcescens*	>0.78	1:2	11.29	1:2
*L. monocytogenes*	>0.78	1:2	5.65	1:4
*S. aureus*	>0.78	1:2	1.41	1:16

*GAE, gallic acid equivalent.*

##### Inhibitory potential (IP)

Table 3 shows the IP of the pulp and seed phenolic extracts of *S. cumini* (L.) Skeels against the evaluated bacteria. The seed phenolic extract had a higher inhibitory activity. It is noteworthy that even at the lowest concentration of 2.82 mg GAE/g of seed (1:8 phenolic extract dilution), there was inhibition of up to four logs for *E. coli*, *Salmonella* Typhimurium and *L. monocytogenes* (Table 3). In a concentration of 11.29 mg GAE/g of the seed (1:2 phenolic extract dilution), all bacteria were completely inhibited, resulting in no growth at the lowest dilution evaluated (Table 3). In addition, *S. aureus* showed higher sensitivity to seed phenolic extract, since this bacterium was completely inhibited in a concentration of 1.41 mg GAE/g of seed (1:16 phenolic extract dilution) (Table 3).

**TABLE 3:** Inhibitory potential (IP) of the pulp and seed phenolic extracts of *S. cumini* (L.) Skeels against the evaluated bacteria.

**Bacteria**	**Phenolic extract**					
	**Pulp**			**Seed**		
	**Concentration (mg GAE/g of pulp)**	**Extract dilution**	**IP**	**Concentration (mg GAE/g of seed)**	**Extract dilution**	**IP**
*E. coli*	0.78	1:2	7.07	11.29	1:2	>8.00
	0.39	1:4	1.60	5.65	1:4	6.86
	0.19	1:8	1.49	2.82	1:8	3.90
*P. aeruginosa*	0.78	1:2	3.50	11.29	1:2	>8.00
	0.39	1:4	1.50	5.65	1:4	6.09
	0.19	1:8	0.97	2.82	1:8	2.22
*Salmonella* Typhimurium	0.78	1:2	3.82	11.29	1:2	>8.00
	0.39	1:4	1.58	5.65	1:4	>8.00
	0.19	1:8	1.07	2.82	1:8	4.18
*L. monocytogenes*	0.78	1:2	3.83	11.29	1:2	>8.00
	0.39	1:4	1.94	5.65	1:4	>8.00
	0.19	1:8	0.81	2.82	1:8	4.11
*S. aureus*	0.78	1:2	3.89	11.29	1:2	>8.00
	0.39	1:4	0.76	5.65	1:4	>8.00
	0.19	1:8	N.I.	2.82	1:8	>8.00
				1.41	1:16	>8.00

GAE, gallic acid equivalent. IP, inhibitory potential equal to 1 indicates 10-fold inhibition compared to the control, due to logarithmic scale; N.I., no inhibition. *We considered no inhibition when bacterial populations between treatment and control differed by less than twofold (IP ¡ 0.301).

#### Anti-QS activity of Phenolic Extracts

The anti-QS activity evaluations by measuring swarming motility and biofilm formation assays were performed only with the pulp extract, because the seed extract could not be properly dissolved in LB medium under the conditions used in these assays. The reason for this is that when the seed extract was added to broth (either LB or BHI), there was some kind of emulsion formed, making any kind of Optical Density reading unattainable. It is important to point out that for the antibacterial assays described previously, the assay could be finished because we counted the CFUs. Therefore, the problem with solubility of the compound was only an issue when performing the QS assays. For instance, when we tried to do the swarming assay, the medium would not solidify in the presence of the seed extract. In the violacein tests, we extract, purify and perform the reading, so the emulsion did not interfere in the final OD reading, allowing the execution of the test for active film added with phenolic seed extract.

##### Swarming motility

**FIGURE 1:**
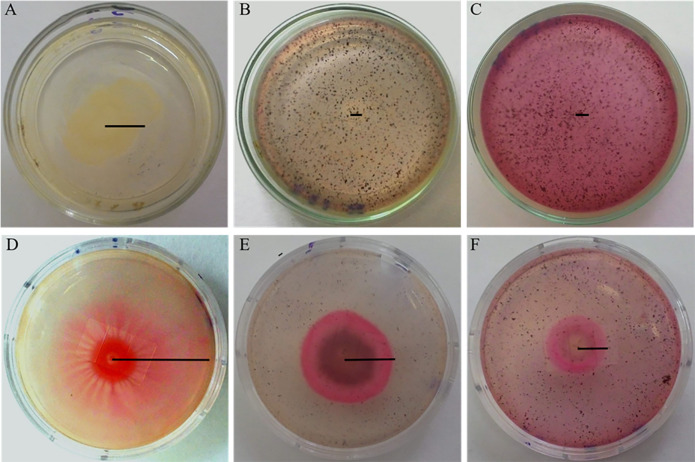
Swarming motility of *A. hydrophila* **(A–C)** and *S. marcescens* **(D–F)** in semi-solid LB agar 0.5% (w/v) without phenolic extract **(A,D)** and with addition of 0.19 **(B,E)** and 0.39 mg GAE/g **(C,F)** of pulp phenolic extract of *S. cumini* (L.) Skeels. Black line indicates the radius of the colony.

Figures 1B,C show the complete inhibition of the swarming motility of *A. hydrophila* by the pulp phenolic extract of jambolan in the two tested concentrations, in comparison with the control giving a swarm of 27 mm (Figure 1A). Figure 1D shows a swarm of 38 mm for *S. marcescens* cultivated in LB semi-solid agar 0.5% (w/v) without pulp phenolic extract of *S. cumini* (L.) Skeels. We observed that with sub-MIC of the pulp phenolic extract, 0.19 and 0.39 mg GAE/g of pulp, there was a clear inhibition of the swarm zone and motilities were 23 and 15 mm, respectively (Figures 1E,F). Even at a low concentration, the pigment present in the extract turned the agar slightly pink (Figure 1E).

##### Biofilm formation

**FIGURE 2:**
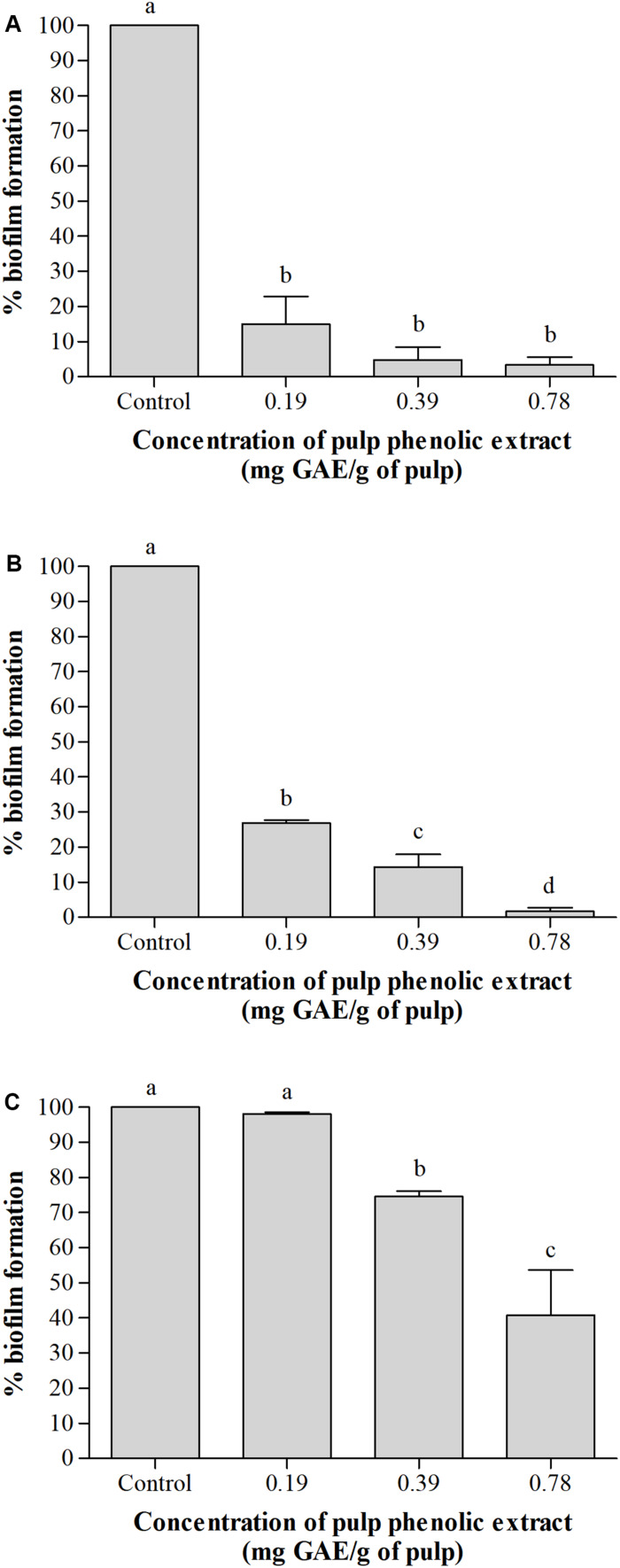
Percentage (%) of biofilm formation by *A. hydrophila* **(A)**, *E. coli* **(B)**, and *S. marcescens* **(C)** in absence and presence of pulp phenolic extract of *S. cumini* (L.) Skeels at sub-MIC of 0.19, 0.39, and 0.78 mg GAE/g of pulp. Means followed by different letters differ statistically (*p* < 0.05).

Figure 2 shows the percentage of biofilm formation by the tested bacteria in the presence of pulp phenolic extract of *S. cumini* (L.) Skeels compared to the control without phenolic extract, which was considered 100%. The pulp phenolic extract reduced biofilm formation by *A. hydrophila* and *E. coli* in all concentrations tested (*p* < 0.05) (Figures 2A,B), while *S. marcescens* biofilm was reduced at the higher tested concentrations (*p* < 0.05) (Figure 2C).

##### Violacein production

**FIGURE 3:**
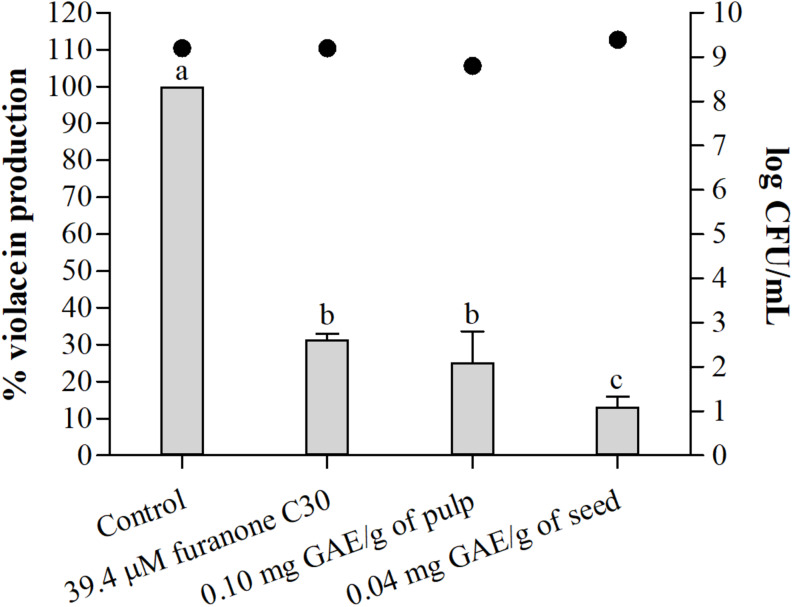
Percentage (%) of violacein production by *C. violaceum* ATCC 12472 in absence and presence of furanone C30, sub-MIC of pulp, and seed phenolic extracts of *S. cumini* (L.) Skeels. Bars represent % violacein production and points represent bacterial population in log CFU/mL. Means followed by different letters differ statistically (*p* < 0.05).

Figure 3 shows the percentage of violacein production by *C. violaceum* according to the presence of pulp and seed phenolic extracts of *S. cumini* (L.) Skeels compared to the control without extract and with furanone C30. There was no effect on the microbial growth (Figure 3). The pulp phenolic extract inhibited violacein production by up to 75% (*p* < 0.05) (Figure 3). The seed phenolic extract showed a remarkable 87% reduction in violacein production compared with 68% for furanone C30, which is the control for inhibition in this assay (Figure 3).

### Active Films Incorporating Pulp and Seed Phenolic Extracts of *S. cumini* (L.) Skeels

#### *In vitro* Antibacterial Activity of Active Films

Table 4 shows the IP of the active films incorporating pulp and seed phenolic extracts of *S. cumini* (L.) Skeels. Two 2-cm diameter discs of film were used for each treatment, equivalent to 2.00 × 10^–4^ mg GAE/g of pulp and 7.28 × 10^–4^ mg GAE/g of seed. Both active films inhibited most of the tested bacteria (Table 4). Nevertheless, the film added with pulp phenolic extract was mostly inhibitory against *L. monocytogenes* and *S. aureus* (Table 4).

**TABLE 4:** Inhibitory Potential (IP) of the active films incorporating pulp and seed phenolic extracts of *S. cumini* (L.) Skeels against the evaluated bacteria.

**Bacteria**	**Phenolic extract**			
	**Pulp**		**Seed**	
	**Concentration (mg GAE/g of pulp)**	IP*	**Concentration (mg GAE/g of seed)**	IP*
*E. coli*	2.00 × 10^–4^	N.I.	7.28 × 10^–4^	N.I.
*P. aeruginosa*	2.00 × 10^–4^	N.I.	7.28 × 10^–4^	N.I.
*P. fluorescens*	2.00 × 10^–4^	0.46	7.28 × 10^–4^	1.50
*Salmonella* Typhimurium	2.00 × 10^–4^	N.I.	7.28 × 10^–4^	0.48
*L. monocytogenes*	2.00 × 10^–4^	2.06	7.28 × 10^–4^	0.60
*S. aureus*	2.00 × 10^–4^	1.62	7.28 × 10^–4^	1.62

GAE, gallic acid equivalent; IP, inhibitory potential equal to 1 indicates 10-fold inhibition, due to logarithmic scale; N.I., no inhibition. * We considered no inhibition when bacterial populations between treatment and control differed by less than twofold (IP < 0.301).

#### *In vitro* Anti-QS Activity of Active Films

##### Violacein Production

**FIGURE 4:**
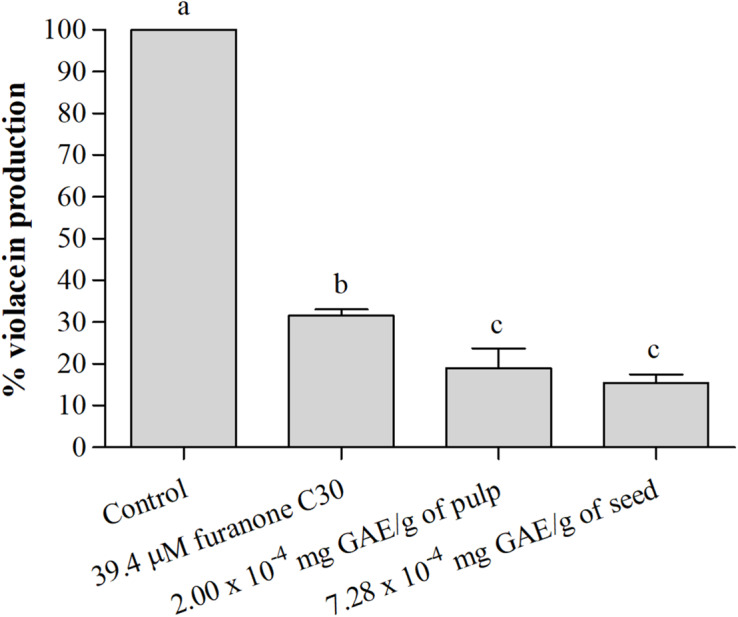
Percentage (%) of violacein production by *C. violaceum* ATCC 12472 in the absence and presence of the active films incorporating pulp and seed phenolic extracts of *S. cumini* (L.) Skeels. Means followed by different letters differ statistically (*p* < 0.05).

Figure 4 shows the percentage of violacein production by *C. violaceum* in the presence of active films. The films with the pulp and seed phenolic extracts showed a significant (*p* < 0.05) reduction of violacein production compared to the negative control (Figure 4). The results demonstrate that film with pulp and seed phenolic extract presented greater reductions than the film with furanone C30, a known QS inhibitor (Figure 4).

### *In silico* Analysis of Compounds Found in Pulp and Seed Extracts of *S. cumini* (L.) Skeels

#### Molecular Docking With Structures of CviR Protein of *C. violaceum* ATCC 12472

Supplementary Table S1 shows the compounds found in pulp and seed extracts of *S. cumini* (L.) Skeels obtained by different solvents and different identification methods by Faria et al. (2011), Tavares et al. (2016, 2017), Azima et al. (2017), and Gajera et al. (2017). A total of 111 different compounds have been found in the pulp extracts and, to those, seven compounds were also present in the seed extracts, such as caffeic acid, catechin, chlorogenic acid, ellagic acid, ferulic acid, gallic acid and quercetin (Supplementary Table S1). However, only 47 of these compounds had the structures deposited in PubChem and could be used for *in silico* studies (Table 5).

**TABLE 5:** Molecular docking of CviR structures of *C. violaceum* ATCC 12472 with phenolic compounds found in pulp and seed extracts of *S. cumini* (L.) Skeels.

**Extract**	**Compound**	**PubChem CID**	**3QP6**				**3QP8**	
			**Binding residue**	**Score**	**Rank**	**Binding residue**	**Score**	**Rank**
n.a.	3-OH-C10-HSL	71353010	Y80, W84, Y88, D97, S155	−85.11	Y80, W84, Y88, D97, S155	−81.54	1
n.a.	C10-HSL	11644562	Y80, W84, D97, S155	−82.21	2	Y80, W84, S155	−81.28	2
Pulp	Gallotannins	452707	Q70, S82, W84, S89, N92	−56.34	8	M72, N77, S82	−75.36	3
Pulp	Catechin 3-O-gallate	5276454	Y88, S89, N92	−57.13	7	Y88, N92, A94	−69.27	4
n.a.	C6-HSL	3462373	W84, Y80, D97, S155	−66.92	3	Y80, W84, S155	−66.69	5
Pulp	Rutin	5280805	Y80, W84, L85, Y88, S89, D97, T140, S155	−19.8	44	M72, Y88, N92, A94	−62.87	6
Pulp and seed	Chlorogenic acid	1794427	W84, S89	−64.41	4	W84, S89	−62.26	7
Pulp	Dihydroquercetin	471	Y88, S155	−61.8	5	L85, Y88, S155	−61.82	8
Pulp	Epicatechin 3-O-gallate	107905	S89, N92	−44.49	21	M72, E73, Y88, S89, N92, A94	−61.27	9
Pulp	Epicatechin	72276	Y88, D97	−54.34	10	Y88, D97, M135	−60.76	10
Pulp	Gallocatechin 3-O-gallate	199472	Y88, S89, N92	−46.77	17	Y88, A94	−60.32	11
Pulp	6-methyldihydroquercetin	182026	L85, D97	−55.21	9	L85, D97	−59.15	12
Pulp	Epigallocatechin 3-O-gallate	65064	L85, S89, N92	−42.62	24	M72, E73, Y88, A94	−58.81	13
Pulp	Peonidin 3,5-diglucoside	44256843	Y80, W84, S89, S155	−1.26	47	M72, V75, Y88, S89, N92, A94	−58.37	14
Pulp	Cyanidin 3-glucoside	12303203	Y88, S89, N92	−52.46	12	Y88, S89	−57.35	15
Pulp	Kaempferol	5280863	M135, S155	−58.54	96	Y80, M135, S155	−56.46	16
Pulp and seed	Quercetin	5280343	M135, S155	−52.95	11	M135, S155	−53.87	17
Pulp	Delphinidin 3-glucoside	443650	Y88	−44.57	20	Y88, S89	−53.44	18
Pulp	Delphinidin 3,5-diglucoside	10100906	Y80, S89, T140, S155	−6.3	45	V75, N77, S89, N92	−53.39	19
Pulp	Petunidin 3-glucoside	443651	S89	−43.63	23	L85, Y88, S89, N92	−52.67	20
Pulp	Cyanidin 3,5-diglucoside	441688	Y80, L85, T140, S155	−6.04	46	V75, Y88, N92, A94	−52.15	21
Pulp	Epigallocatechin	72277	L85, Y88, S155	−50.9	14	W84, L85, Y88, S155	−52.08	22
Pulp	Malvidin 3,5-diglucoside	44256978	M72, W84, Y88, S89, N92	16.96	48	M72, E73, V75, Y88, S89	−51.92	23
Pulp	Malvidin 3-glucoside	443652	S89	−40.82	26	Y88, S89, A94	−51.69	24
Pulp and seed	Catechin	9064	T140, S155	−52.4	13	Y80, D97, M135, S155	−51.51	25
Pulp	Myricetin 3-glucoside	44259426	Y88, S89	−36.39	30	Y88, S89, N92, A94	−51.27	26
Pulp	Lyricetin 3-rhamnoside	5281673	Y88, S89	−38.57	28	S89	−51.02	27
Pulp	Laricitrin	5282154	W84	−44.09	22	W84	−49.89	28
Pulp	Syringetin	5281953	W84	−42.11	25	W84, S155	−49.27	29
Pulp	Gallocatechin	65084	L85	−50.07	16	L85	−48.86	30
Pulp	Galloyl glucose	124021	W84, D97	−50.28	15	W84	−48.53	31
Pulp	Petunidin 3,5-diglucoside	10151874	L85	−32.86	34	I69, Q70, L85, N92, A94	−48	32
Pulp	Dihydromyricetin	161557	–	–	–	S89	−47.07	33
Pulp	Myricetin	5281672	–	–	–	W84	−46.63	34
Pulp and seed	Ferulic acid	445858	Y80, S155	−46.05	18	Y80, S155	−45.85	35
Pulp	Myricetin 3-glucuronide	44259442	Y88, S89	−32.82	35	Y88, S89, N92	−45.78	36
Pulp and seed	Ellagic acid	5281855	W84	−39.55	27	W84	−45.66	37
Pulp	Laricitrin 3-glucoside	44259475	Y88, S89	−32.29	36	S89, N92, A94	−45.42	38
Pulp and seed	Caffeic acid	689043	M135	−45.04	19	M135	−45.18	39
Pulp	Myricetin 3-galactoside	5491408	Y88, S89, N92	−35.07	32	Y88, A94	−45.02	40
Pulp	Laricitrin 3-galactoside	44259474	Y88	−30.58	37	Y88, S89	−43.36	41
Pulp	Galloyl (+)-hexahydroxydiphenic acid	129733714	W84, D97, Y88, S89, N92	−29.24	39	L85, Y88, S89, N92, A94	−43.09	42
Pulp	Myricetin 3-pentoside	21477996	Y88, S89	−30.17	38	S89	−40.98	43
Pulp	Syringetin 3-glucoside	44259492	S89	−29	40	S89	−40.88	44
Pulp	Vanillic acid	8468	M135, S155	−38.31	29	S155	−38.28	45
Pulp	Valoneic acid	71308296	W84, L85, Y88, D97, S155	−22.87	43	S82, Y88, S89, N92	−37.98	46
n.a.	Furanone C30	10131246	Y80, T140, S155	−34.39	33	W84	−33.6	47
Pulp and seed	Gallic acid	370	S155	−35.29	31	S155	−33.34	48
Pulp	Hexahydroxydiphenic acid	10315050	L85, Y88, S89, A94	−23.52	42	L85, S89	−27.67	49
Pulp	Vescalagin (Castalagin)	168165	I69, Q70, L85, Y88, S89	430.49	49	M72, V75, Y88, S89, A94	212.99	50
Pulp	Syringetin 3-galactoside	44259488	S89, N92	−27.29	41	–	–	–

*n.a., not applicable; –, the compound did not bind to the CviR protein; Rank, binding affinity scale between CviR protein with 3-OH-C10-HSL, C10-HSL, C6-HSL, furanone C30, and 47 compounds found in pulp and seed extracts of *S. cumini* (L.) Skeels based on score value. The color of this scale is a color ramp ranging from dark pink (higher affinity) to dark green (lower affinity) and hyphen for no binding. The main results discussed in the text are shown with gray background.*

Molecular docking of CviR 3QP6 and 3QP8 structures showed that AHLs with ten carbons had the highest binding affinities. The scores were −85.11 and −81.54 for 3-OH-C10-HSL with 3QP6, and 3QP8 −82.21, and −81.28 for C10-HSL with 3QP6 and 3QP8, respectively (Table 5). The C6-HSL also showed high binding affinity for both structures, even though gallotannins and catechin 3-*O*-gallate presented better affinity with 3QP8 (Table 5). These compounds are found in the pulp extracts of *S. cumini* (L.) Skeels and, together with chlorogenic acid and dihydroquercetin, have shown the highest binding affinities for both structures of CviR. Chlorogenic acid was also found in seed extract and presented high binding affinity with both structures. Interestingly, rutin presented high affinity to only the 3QP8 structure (Table 5). It is noteworthy that the binding affinity of these compounds was higher than that of furanone C30 (Table 5). In general, of the 47 evaluated phenolic compounds, only dihydromyricetin, myricetin, and syringetin 3-galactoside did not dock in one of the two evaluated structures (Table 5).

**FIGURE 5:**
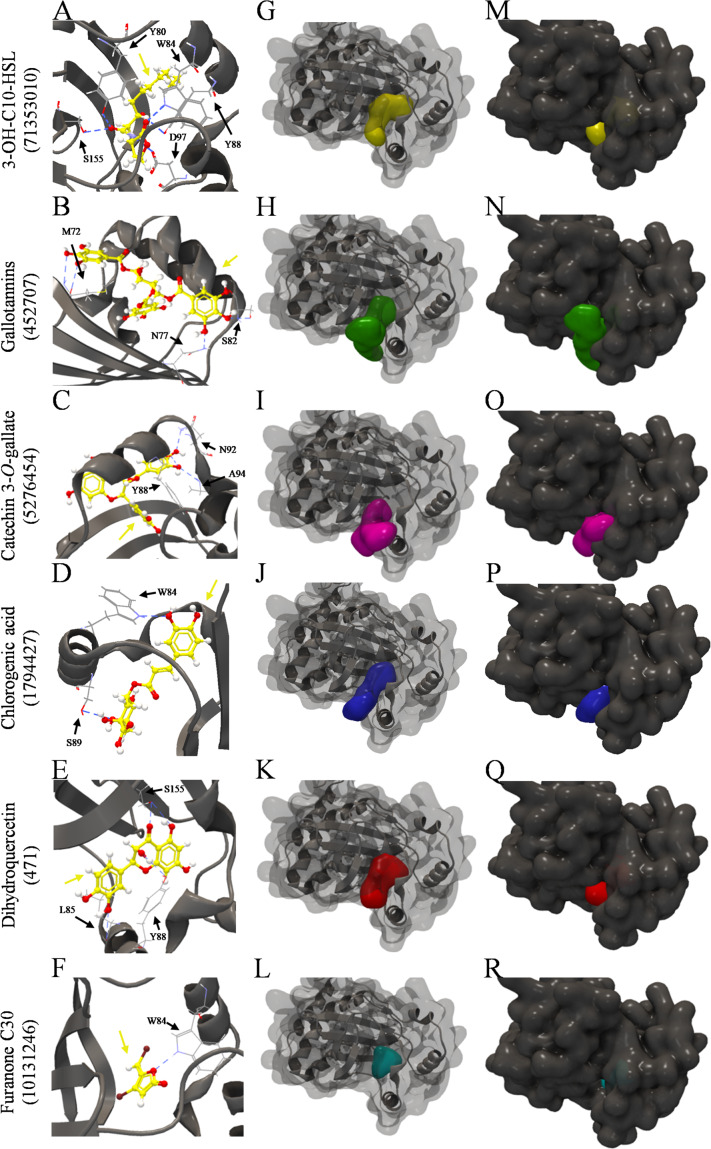
Molecular docking of 3QP8 structure of CviR protein of *C. violaceum* ATCC 12472 with 3-OH-C10-HSL, gallotannins, catechin 3-*O*-gallate, chlorogenic acid, dihydroquercetin, and furarone C30. **(A–F)** Backbone representation of 3QP8 structure with hydrogen bond between the amino acid residues and evaluated compounds, **(G–L)** surface and backbone representations, and **(M–R)** surface representation. Gray surface representation, CviR; yellow surface representation, 3-OH-C10-HSL; green surface representation, gallotannins; pink surface representation, catechin 3-*O*-gallate; blue surface representation, chlorogenic acid; red surface representation, dihydroquercetin; cyan surface representation, furarone C30; gray backbone representation, CviR; black arrow indicates the binding site; yellow arrow, 3-OH-C10-HSL or gallotannins or catechin 3-*O*-gallate or chlorogenic acid or dihydroquercetin or furarone C30; blue dashed line, hydrogen bond.

The inspection of the binding sites showed that 3-OH-C10-HSL bound to Y80, W84, Y88, D97, and S155 residues of the two structures of *C. violaceum* CviR protein (Table 5 and Figure 5A). Chlorogenic acid bound to W84 and S89 residues of the two structures (Table 5 and Figure 5D). On the other hand, gallotannins (Table 5 and Figure 5B), catechin 3-*O*-gallate (Table 5 and Figure 5C), dihydroquercetin (Table 5 and Figure 5E), and furanone C30 (Table 5 and Figure 5F) bound at different amino acid residues in these two structures. The Y88 residue of both structures is a common binding site for 3-OH-C10-HSL and catechin 3-*O*-gallate. The W84 residue is a common binding site for 3-OH-C10-HSL and chlorogenic acid, and Y88 and S155 residues are common binding sites for 3-OH-C10-HSL and dihydroquercetin (Table 5). Gallotannins presented only W84 residue of one of the structures in common with this AHL, and furanone C30 showed Y80, W84, and S155 residues in common (Table 5).

The 3-OH-C10-HSL (Figures 5G,M), dihydroquercetin (Figures 5K,Q), and furanone C30 (Figures 5L,R) were well buried in the pocket of the two CviR structures, unlike gallotannins (Figures 5H,N), catechin 3-*O*-gallate (Figures 5I,O), and chlorogenic acid (Figures 5J,P), which were not fully enclosed in the binding pocket.

## Discussion

In this study, we have evaluated the physicochemical composition, the antioxidant potential and the antimicrobial and anti-QS activities of the extracts obtained from the pulp and the seeds of *S. cumini* (L.) Skeels.

We observed high moisture content in the pulp (83.51%) of *S. cumini* (L.) Skeels (Table 1). According to Chitarra and Chitarra (2005), fruit pulps in general have high moisture content that ranges between 65 and 95%. The pulp had a lipid content of 0.97%, considered hypocaloric, and therefore its consumption should be stimulated in diets with caloric restriction. The pulp had a protein content of 5.62%, which differed from that reported by Vizzotto and Pereira (2008), which was 0.67%. The observed differences could be attributed to changes in the maturation stages, soil type, weather conditions and farming practices (Chitarra and Chitarra, 2005).

The pulp total soluble solids content (12.91°Brix) was higher than that found by Vizzotto and Pereira (2008) for the same fruit (9°Brix). These differences can be attributed to the different maturation stages of fruit and other factors mentioned above (Chitarra and Chitarra, 2005). The fruits of this study were harvested in the mature fruiting season, and it is known that the content of organic acids tends to decrease with the maturation process because of their use as substrate in respiration or conversion to the plant metabolism (Vilas Boas, 2006). The pH value of the pulp was 4.12 and according to Kuskoski et al. (2006), jambolan can be classified as an acidic food.

The concentration of phenolic compounds was higher in the seed than in the pulp of jambolan (Table 1). Studies have shown that phenolic compounds have different properties such as anti-aging, anti-inflammatory, antioxidant, and antiproliferative activities (Manach et al., 2004). Not surprisingly, the seed also presented a higher antioxidant activity (Table 1). According to Ayyanar and Subash-Babu (2012), jambolan seeds are known to be rich in flavonoids, which explains the higher antioxidant activity. It is generally considered beneficial to consume phenolic compound-rich foods (Lin et al., 2016).

Knowing the potential phenolic content of the pulp and the seeds, we then evaluated the antibacterial activity of phenolic extracts prepared from these samples. In the agar diffusion assay, only *S. aureus* was inhibited by the seed phenolic extract. Singh et al. (2016) demonstrated that pulp extracts of *S. cumini* (L.) Skeels had similar results for *S. aureus* in this assay. Alves et al. (2000) evaluated the antimicrobial activity of plants from the Brazilian *cerrado* biome and created a classification scheme according to the inhibition zones: <9 mm, inactive; 9–12 mm, partially active; 13–18 mm, active; >18 mm, very active. Thus, the seed jambolan phenolic extract can be classified as very active against *S. aureus* with an inhibition of 24.5 mm. On the other hand, the other bacteria were not inhibited by the jambolan extracts when using this method.

Thus, we decided to test the antibacterial activity in liquid medium. The MIC of the pulp phenolic extract was higher than 0.78 mg AGE/g of pulp (1:2 phenolic extract dilution), and the MIC values of the seed phenolic extract ranged from 1.41 to 11.29 mg GAE/g of seed (dilutions ranging from 1:2 to 1:256), depending on the bacterium. Even using the most concentrated pulp extract (1:2 dilution), we could not see inhibition, which is likely explained by the low phenolic content of this extract (Table 1). On the other hand, for *S. aureus*, the seed phenolic extract showed inhibition in the dilution 1:16, indicating that this extract might be an appropriate option in the control of *S. aureus* growth. Additional work should be performed with different strains of *S. aureus* in order to confirm this hypothesis. Bag et al. (2012) studied the antimicrobial potential of seeds of *S. cumini* (L.) Skeels using different extraction methods and kill-kinetics assays, finding inhibition in the range of 0.08–0.845 mg of seed extract/mL of DMSO for the tested bacteria such as *S. aureus* ATCC 6538P, *E. coli* ATCC 10536 and *P. aeruginosa* ATCC 15442. These results confirm the potential presented by *S. cumini* (L.) Skeels as a source of antimicrobial compounds.

We have used a third antimicrobial assay referred to as the IP of the phenolic extracts (Alvarez et al., 2012). Both extracts presented IP for all the evaluated bacteria, but the seed phenolic extract was more effective. At 11.29 mg GAE/g of seed, all bacteria were inhibited, resulting in no growth at the limit of detection of the plating method. Once again, *S. aureus* was mostly sensitive to seed phenolic extract, even at the lowest tested concentration. The higher IP of the seed compared to the pulp may be related to its higher content of phenolic compounds (Table 1). According to Xu et al. (2014), as phenolic compounds present a partially hydrophobic nature, they can interact more effectively with the lipid bilayer of bacterial membrane, causing instability and an overflow of cellular content, interfering in nutrient transport and having a greater antibacterial effect.

In addition, we observed that the phenolic extracts showed greater efficacy against Gram-positive bacteria such as *L. monocytogenes* and *S. aureus*. According to Tortora et al. (2012), this could be due to the membrane composition differences among the two bacterial groups. Taguri et al. (2004) studied the relation of flavonoids with antimicrobial activity, inferring that the total rupture of microbial membranes can be a result of the lipophilic flavonoids. It is also suggested that vegetable extracts obtained with organic solvents, as was done here, have the capacity to extract more non-polar molecules, potentializing their antimicrobial activity.

We have analyzed the anti-QS activity of pulp and seed phenolic extracts of *S. cumini* (L.) Skeels in some phenotypes such as swarming motility, biofilm formation and violacein production. The first two were performed only with the pulp phenolic extract, since the seed extract could not be properly dissolved under the conditions used in these assays, as previously explained. At sub-MIC of pulp phenolic extract, there was effective inhibition of swarming motility of *A. hydrophila* and *S. marcescens* compared to the control. At a concentration of 0.39 mg AGE/g of fruit, there was inhibition of prodigiosin production, a QS regulated reddish color pigment characteristic of *S. marcescens* (Packiavathy et al., 2014). Biofilm formation by *A. hydrophila*, *E. coli* and *S. marcescens* was reduced by the pulp phenolic extract. The reduction in swarming motility and biofilm formation could be associated with the presence of phenolic compounds, which may affect microbial appendages like fimbriae and flagella, essential for the adhesion/fixation of bacteria to surfaces, and, consequently, to form biofilms (Jamal et al., 2018).

The seed phenolic extract also showed a significant reduction in violacein production compared to the control. The pulp phenolic extract also inhibited violacein production. Vasavi et al. (2013) showed inhibition of violacein production by ethanolic extract of *S. cumini* (L.) Skeels leaves in *C. violaceum* without altering microbial growth. They used the leaf powder of jambolan dissolved in DMSO at concentrations of 0.25 to 1 mg/mL, where 1 mg of leaf powder/mL of DMSO completely inhibited violacein production. Thus, other parts of *S. cumini* (L.) Skeels also have the potential to inhibit QS. The inhibition of violacein production suggest that phenolic compounds found in many fruit extracts have the capacity to inhibit QS, corroborating our previous findings on swarming motility and biofilm formation.

As a means to test a practical application of the jambolan extracts, we have incorporated the pulp phenolic extract into cellulose acetate films for further analyses. The film was more effective against Gram-positive bacteria such as *L. monocytogenes* and *S. aureus*. As formerly mentioned, membrane composition and stability upon interaction with phenolic compounds may be more pronounced in Gram-positives (Tortora et al., 2012; Xu et al., 2014).

No studies have been found evaluating the antimicrobial activity of films incorporating extracts of *S. cumini* (L.) Skeels. Dannenberg et al. (2017) evaluated the antimicrobial activity of films incorporating essential oil of rose pepper (*Schinus terebinthifolius* RADDI), which showed inhibitory activity against *L. monocytogenes*, *S. aureus*, *E. coli* and *Salmonella* Typhimurium, even though inhibition of Gram-negative bacteria was only seen at higher concentrations. Since natural extracts usually contain several active molecules with distinct mechanisms of action and cellular targets, it is more difficult for bacteria to develop resistance (Burt, 2004). Thus, natural extracts can be considered as an alternative means to fight bacterial resistance.

Vasavi et al. (2013) evaluated the effect of different leaf extracts of *S. cumini* (L.) Skeels against *C. violaceum* strains and found that ethyl acetate fractions presented the highest inhibition against violacein production. Their results corroborate our findings in which the films incorporating the phenolic extracts of pulp and seed showed inhibition of violacein production by *C. violaceum* ATCC 12472.

We extensively searched the literature for compounds that are commonly found in pulp and seed extracts of *S. cumini* (L.) Skeels (Faria et al., 2011; Tavares et al., 2016, 2017; Azima et al., 2017; Gajera et al., 2017). We then performed *in silico* molecular docking between these compounds and the 3QP6 and 3QP8 structures of the QS receptor CviR protein of *C. violaceum* ATCC 12472. The 3QP6 structure was crystalized with C6-HSL, which is an inhibitor of QS in this strain, and the 3QP8 structure was crystalized with the inducer C10-HSL (Chen et al., 2011). Molecular docking was also performed with 3-OH-C10-HSL, which is the inducer most abundantly synthesized by *C. violaceum* ATCC 12472 (Morohoshi et al., 2008) and with furanone C30, a well-known inhibitor (Oliveira et al., 2016). All phenolic compounds found in the pulp and seed extracts presented lower binding affinity to CviR than AHLs with ten carbons in the side chain. However, several compounds presented similar binding affinities to the C6-HSL inhibitor and many had higher affinity than furanone C30, especially gallotannins, catechin 3-*O*-gallate, chlorogenic acid, and dihydroquercetin.

Chlorogenic acid and dihydroquercetin presented common binding sites to 3-OH-C10-HSL (Table 5). Wang et al. (2019) showed that chlorogenic acid inhibited violacein production, swarming motility and biofilm formation by *C. violaceum* ATCC 12472, as well as biofilm formation, swarming motility and rhamnolipid production by *P. aeruginosa* PAO1 and pyocyanin production in PA14. Other genes related to QS in *P. aeruginosa* PAO1 were also downregulated by chlorogenic acid, in addition to *in silico* binding to LasR and RhlR QS receptors (Wang et al., 2019). Vandeputte et al. (2011) showed that dihydroquercetin, also called taxifolin, at 4 mM reduced violacein production by *C. violaceum* CV026 as well the production of pyocyanin and elastase in *P. aeruginosa* PAO1. These authors showed that taxifolin reduced the expression of several QS-controlled genes, such as *lasI*, *lasR*, *rhlI*, *rhlR*, *lasA*, *lasB*, *phzA1* and *rhlA* in *P. aeruginosa* PAO1. Quecán et al. (2019) have shown that violacein production by *C. violaceum* ATCC 12472 was inhibited in the presence of 15.6 μg/mL of quercetin. These authors have also shown that quercetin was able to accommodate in the pocket of these CviR structures. Altogether, these results indicate that the compounds found in the pulp and seed extracts of *S. cumini* (L.) Skeels may compete individually or synergistically with AHLs for the CviR binding site. This hypothesis is reinforced by the results of the inhibition of violacein production by pulp and seed phenolic extracts and in the active film. It would be interesting to follow up with additional studies including isolated compounds as well as the evaluation of their combined action upon different QS biosensor models.

## Conclusion

**FIGURE 6:**
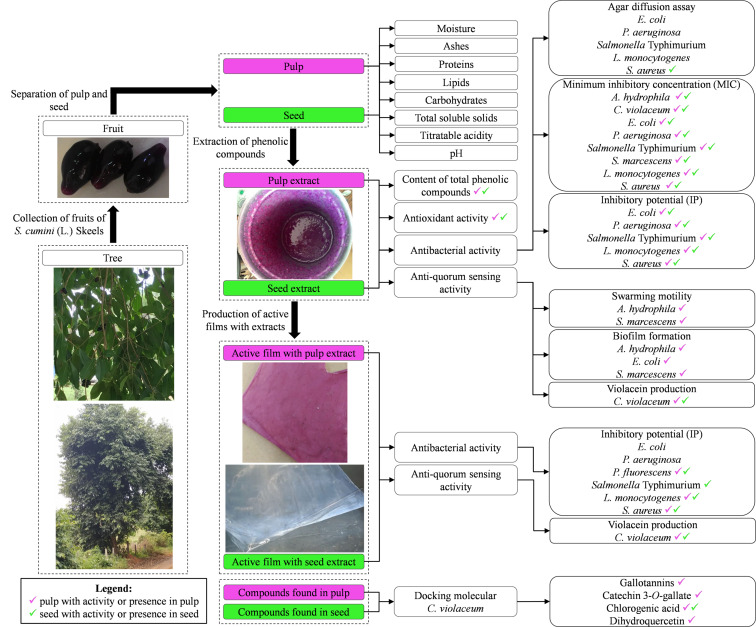
Experimental design used in this study with the main findings.

Figure 6 is a graphical summary of this study. The seed phenolic extract of *S. cumini* (L.) Skeels presented higher content of total phenolic compounds and antioxidant activity than pulp phenolic extract. Both extracts showed antibacterial activity against *A. hydrophila*, *C. violaceum*, *E. coli*, *P. aeruginosa*, *Salmonella* Typhimurium, *S. marcescens*, *L. monocytogenes*, and *S. aureus*. Additionally, the pulp phenolic extract inhibited swarming motility and biofilm formation by *A. hydrophila*, *E. coli*, and *S. marcescens* at sub-MIC concentrations. Seed phenolic extract showed greater anti-QS activity against *C. violaceum* compared to the pulp phenolic extract. The films incorporating both phenolic extracts were inhibitory to most evaluated bacteria, especially *P. fluorescens, L. monocytogenes* and *S. aureus*. *In silico* analysis by molecular docking showed that the compounds commonly found in the pulp and seed extracts of jambolan have affinities to the *C. violaceum* CviR protein and therefore may interfere with QS activity, particularly chlorogenic acid and dihydroquercetin. Taken together, these results show that pulp and seed of *S. cumini* (L.) Skeels are good sources of antibacterial, antibiofilm and anti-QS compounds. Jambolan can be used in the development of natural preservatives and applied in antibacterial active films.

## Data Availability Statement

All datasets generated for this study are included in the article/Supplementary Material.

## Author Contributions

All authors listed have made a substantial, direct and intellectual contribution to the work, and approved it for publication.

## Conflict of Interest

The authors declare that the research was conducted in the absence of any commercial or financial relationships that could be construed as a potential conflict of interest.

## Footnotes

1 http://reflora.jbrj.gov.br/reflora/herbarioVirtual/ConsultaPublicoHVUC/ConsultaPublicoHVUC.do

2 http://www.rcsb.org/pdb/home/home.do

3 https://www.qiagenbioinformatics.com/

## Supplementary Material

TABLE S1 Compounds found in pulp and seed extracts of *S. cumini* (L.) Skeels.
